# NOTCH3 inactivation increases triple negative breast cancer sensitivity to gefitinib by promoting EGFR tyrosine dephosphorylation and its intracellular arrest

**DOI:** 10.1038/s41389-018-0051-9

**Published:** 2018-05-25

**Authors:** Giulia Diluvio, Francesca Del Gaudio, Maria Valeria Giuli, Giulia Franciosa, Eugenia Giuliani, Rocco Palermo, Zein Mersini Besharat, Maria Gemma Pignataro, Alessandra Vacca, Giulia d’Amati, Marella Maroder, Claudio Talora, Carlo Capalbo, Diana Bellavia, Saula Checquolo

**Affiliations:** 1 Department of Molecular Medicine, Sapienza University, Rome, Italy; 2 Department of Cell and Molecular Biology, Karolinska Institutet, 17177 Stockolm, Sweden; 3 Novo Nordisk Foundation Center for Protein Research, University of Cophenagen, København, Denmark; 4 Center for Life Nano Science@Sapienza, Istituto Italiano di Tecnologia, Rome, Italy; 5 Department of Radiological, Oncological and Pathological Sciences, Sapienza University, Rome, Italy; 6 Department of Experimental Medicine, Sapienza University, Rome, Italy; 7 Department of Medico-Surgical Sciences and Biotechnology, Sapienza University, Latina, Italy

## Abstract

Notch dysregulation has been implicated in numerous tumors, including triple-negative breast cancer (TNBC), which is the breast cancer subtype with the worst clinical outcome. However, the importance of individual receptors in TNBC and their specific mechanism of action remain to be elucidated, even if recent findings suggested a specific role of activated-Notch3 in a subset of TNBCs. Epidermal growth factor receptor (EGFR) is overexpressed in TNBCs but the use of anti-EGFR agents (including tyrosine kinase inhibitors, TKIs) has not been approved for the treatment of these patients, as clinical trials have shown disappointing results. Resistance to EGFR blockers is commonly reported. Here we show that Notch3-specific inhibition increases TNBC sensitivity to the TKI-gefitinib in TNBC-resistant cells. Mechanistically, we demonstrate that Notch3 is able to regulate the activated EGFR membrane localization into lipid rafts microdomains, as Notch3 inhibition, such as rafts depletion, induces the EGFR internalization and its intracellular arrest, without involving receptor degradation. Interestingly, these events are associated with the EGFR tyrosine dephosphorylation at Y1173 residue (but not at Y1068) by the protein tyrosine phosphatase H1 (PTPH1), thus suggesting its possible involvement in the observed Notch3-dependent TNBC sensitivity response to gefitinib. Consistent with this notion, a nuclear localization defect of phospho-EGFR is observed after combined blockade of EGFR and Notch3, which results in a decreased TNBC cell survival. Notably, we observed a significant correlation between *EGFR* and *NOTCH3* expression levels by in silico gene expression and immunohistochemical analysis of human TNBC primary samples. Our findings strongly suggest that combined therapies of TKI-gefitinib with Notch3-specific suppression may be exploited as a drug combination advantage in TNBC treatment.

## Introduction

Triple-negative breast cancer (TNBC), which lacks estrogen receptor (ER), progesterone receptor, and human epidermal growth factor 2 receptor (HER2), accounts for about 15–20% of breast cancers and represents the most aggressive breast cancer (BC) subtype^1^. To date, no molecularly targeted agents have been approved for TNBC, leaving to the conventional chemotherapy the role of primary option for systemic treatment. Although TNBC-bearing patients better respond to current chemotherapy than do non-TNBC ones, patients with TNBC experience a more rapid relapse evolving as metastatic disease. For this reason, this BC subtype suffers from the poorest prognosis^1^. Therefore, targeted therapeutic strategies for TNBC are urgently needed.

The overexpression of the tyrosine kinase receptor epidermal growth factor receptor (EGFR) is a hallmark of TNBC (45–70%) and exhaustive gene expression profiling has identified several EGFR-associated poor prognostic signatures^2^. Anti-EGFR therapies, including tyrosine kinase inhibitors (TKIs) and monoclonal antibodies, have been developed and are already available for treatment of different cancers such as non-small cell lung cancer (NSCLC) and colorectal cancer, making EGFR inhibitors an attractive option for TNBC therapy^3^. Unfortunately, no EGFR inhibitory therapies are currently approved for BC treatment, including TNBC, as results from clinical trials are disappointing^4^. This limited clinical activity is often due to the existence of compensatory pathways that confer resistance to EGFR inhibition, thus allowing continued cancer cell growth and survival^5–7^.

Notch signaling dysregulation is often associated with tumor transformation^8^, including the TNBC pathogenesis and progression^9–11^. In particular, TNBCs show Notch3 amplification and overexpression^12,13^, and Notch3 knockdown has been shown to reduce the proliferation of ErbB2-negative breast tumor cells^9,14^. More recently, these data have been strongly supported by Choy et al.^15^ who demonstrated that constitutive Notch3 signaling can drive an oncogenic program in a subset of TNBCs, thus suggesting that Notch3 activity (and not others Notch paralogues) may be clinically relevant in this BC subtype. There is a growing body of evidence that Notch hyperactivation or mutation results in several events that enable BC cells to become resistant to targeted treatments through different mechanisms^16,17^, thus suggesting that the inactivation of Notch signaling could be a potential therapeutic approach for overcoming resistance to drugs^7^. Interestingly, more recently, it has been demonstrated that Notch3 pathway is strongly involved in the stroma-mediated expansion of therapy-resistant TNBC cells^18^.

Notch-EGFR interplay occurs in different cellular contexts^19,20^, including BC^16^, raising the possibility that Notch signaling could be involved in the above mentioned resistance to EGFR inhibition. Arasada et al.^21^ first reported that the EGFR inhibition by erlotinib treatment is able to activate Notch signaling in human lung cancer, resulting in an enriched stem cell-like populations in a Notch3, but not Notch1-dependent manner. In TNBC, it has been demonstrated that combined Notch-EGFR pathway inhibition is a rational treatment strategy for this type of tumors^22^. Pan-Notch inhibition using γ-secretase inhibitor (GSI) treatment supports this conclusion. Unfortunately, the use of GSIs fails to distinguish the particular Notch receptor driving growth, besides eliciting severe side effects.

Here we analyze the effects of a selective Notch3 inhibition in the response to gefitinib (GEF) treatment of resistant TNBC cells. We show that Notch3 (but not Notch1) depletion enhances the therapeutic target activity of the EGFR, by inducing its dephosphorylation via protein tyrosine phosphatase H1 (PTPH1), finally leading to an increased TNBC sensitivity to TKI-GEF.

## Results

### Notch3-EGFR correlation in primary TNBC samples

**Fig. 1:**
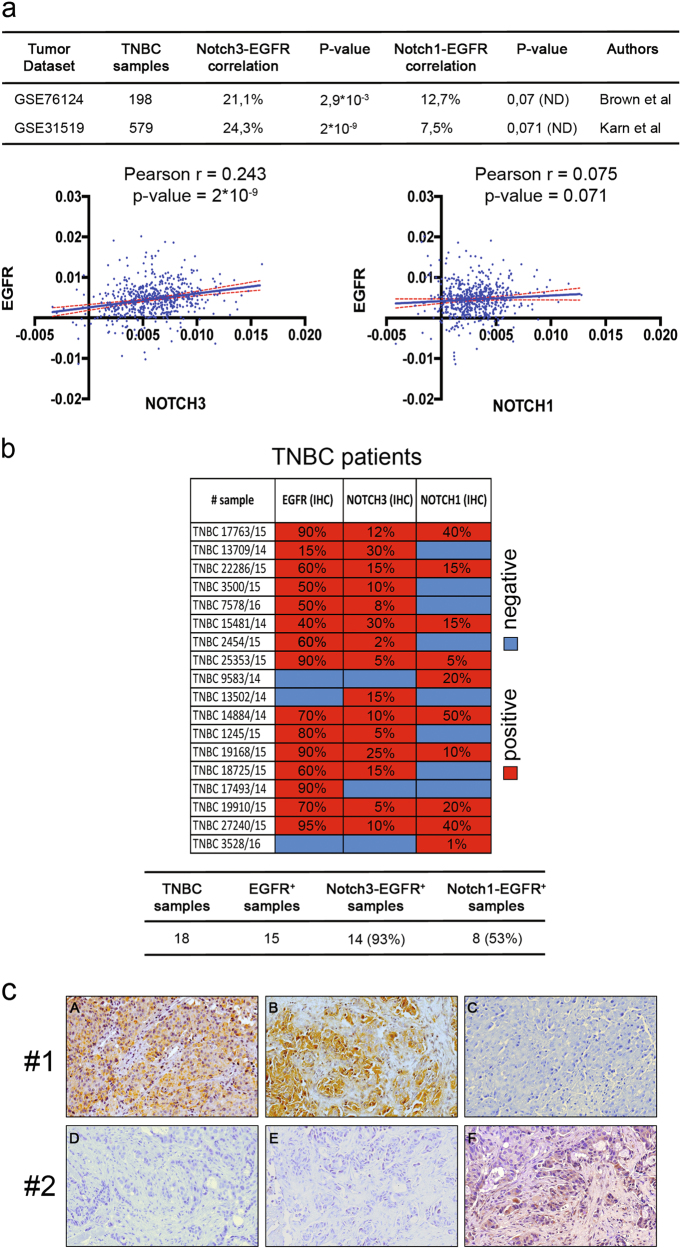
Notch3 and EGFR levels correlate in TNBC primary samples. **a** Upper panel: summary of the *NOTCH3*-*EGFR* and *NOTCH1*-*EGFR* gene expression levels correlation obtained by an in silico analysis from two TNBC tissue arrays (GSE76124 and GSE31519). Lower panels: representative graphs showing correlation between *NOTCH3* (left) or *NOTCH1* (right) and *EGFR* gene expression levels from GSE31519 dataset in a cohort of 579 TNBC patients. In both graphs, each dot corresponds to one patient and the expression value of *NOTCH3*, *NOTCH1*, and *EGFR* is given in log2 scale after normalizing data with justRMA algorithm normalization. The *X*–*Y* axis represent *NOTCH3* (left) or *NOTCH1* (right) and *EGFR* (both) expression levels, respectively. The index Pearson’s R indicated expresses the linear relation between paired samples and *P*-values were calculated using Student’s *T*-test, as described in Material and Methods section. **b** Upper panel: heatmap representing the protein levels of EGFR, Notch3, and Notch1 obtained by immunohistochemical analysis (IHC) in a cohort of 18 TNBC patients. The colors represent positive (red) or negative (blue) protein levels according to protein expression cutoff (see Materials and Methods section). Lower panel: summary of the Notch3-EGFR and Notch1-EGFR protein expression levels correlation showing percentage of each category calculated on the precedent category of patients. **c** Pattern of immunostaining in two different cases of TNBC. In case 1 (upper panels), there is a strong and diffuse staining of neoplastic cells both for EGFR (A) and Notch3 (B), whereas Notch1 is completely negative (C). In case 2 (lower panels), the neoplastic cells are negative for both EGFR (D) and Notch3 (E), whereas Notch1 (F) shows a weak positivity in about 20% of the cells

To deepen the understanding of the possible Notch3-EGFR crosstalk in TNBC context, we first performed an in silico analysis of the *NOTCH3* and *EGFR* gene expression levels in two cohorts of TNBC patients, collectively consisting of 777 individuals^23–26^ (Fig. 1a). The summary of the obtained results (Fig. 1a, upper panel) highlights a direct correlation between *EGFR* and *NOTCH3* gene expression levels in both datasets analyzed, while a weaker correlation between *EGFR* and *NOTCH1* is observed. This is also evident by the graphs included in the Fig. 1a (lower panels), representative of the larger dataset. These data indicate that in a consistent proportion of TNBC-bearing patients (about 23%) the presence of *EGFR* coexists with *NOTCH3* gene expression, allowing us to hypothesize a possible direct relationship between EGFR and Notch3 at the protein level in TNBC. To test this hyphotesis, we then analyzed the pattern of immunohistochemical expression of Notch3, Notch1, and EGFR in tissue samples of 18 human TNBCs. In the majority of cases (15/18), we found EGFR positivity in neoplastic cells. Notch3 is expressed in a higher percentage of EGFR-positive tumors as compared with Notch1 (93% vs. 53%) (Fig. 1b, lower panel). Figure 1c (case 1) shows an example of TNBC tumor expressing both EGFR (panel a) and Notch3 (panel b) but not Notch1 (panel c), representative of 6 out of 15 TNBC EGFR^+^ tissue samples analyzed. Interestingly, two out of three TNBC samples not expressing EGFR are also Notch3 negative but express Notch1 (Fig. 1c, case 2), thus reinforcing the relevant Notch3-EGFR direct correlation in this cancer subtype.

### Notch3 inhibition by siRNA sensitizes TNBC cells to EGFR-TKI-GEF treatment

To examine whether Notch3 could be involved in the mechanism of resistance to EGFR TKI, we first selected a group of TNBC cells expressing EGFR at various levels and known to be EGFR-TKI-resistant cells^27,28^, and then we analyzed the expression of both Notch3 and Notch1 proteins (Supplementary Figure S1a). Almost all TNBC cells expressed activated Notch1 and/or Notch3 protein (N3_IC_), thus confirming the hyperactivation of Notch signaling observed in this BC subtype^14^, mainly involving the upregulation of N3_IC_ expression, as it appears at undetectable levels in MCF10A, a normal immortalized mammary epithelial cell line. This also occurs in MDA-MB-453 cells, which express lower EGFR expression (data not shown) (Supplementary Figure S1a).

**Fig. 2:**
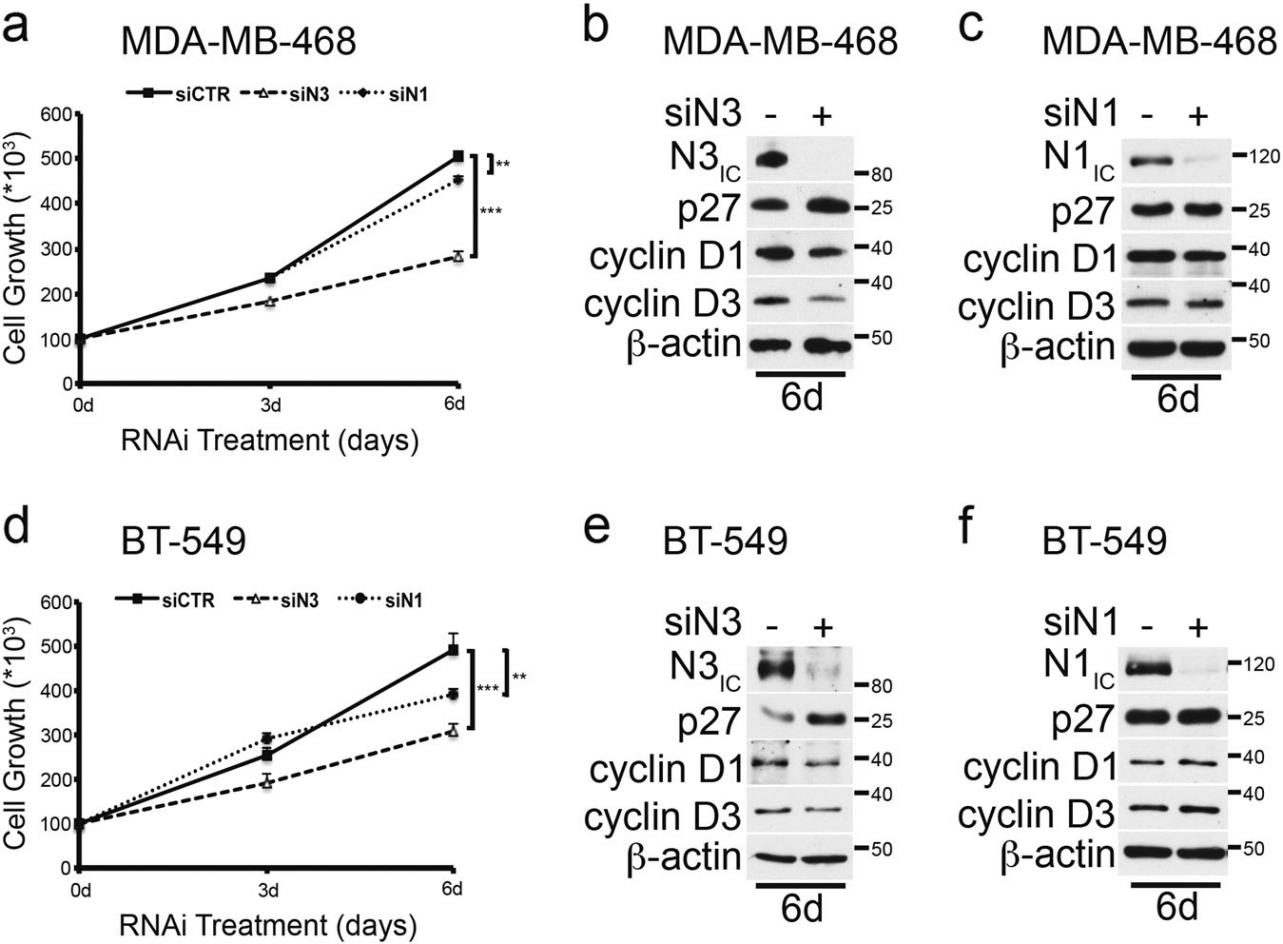
Notch3 downregulation by siRNA affects TNBC cells survival. **a**, **d** Analysis of cell growth after 0–3–6 days of Notch3 and Notch1 silencing in **a** MDA-MB-468 and **d** BT-549 cells. **b**, **c** Whole cell extracts from **a** MDA-MB-468 or **d** BT-549 cells at 6 day of silencing were used for western blot against Notch3 (N3_IC_) and Notch1 (N1_IC_), to control the efficiency of the **b**, **e** Notch3 and **c**, **f** Notch1 silencing, respectively. Extracts were then immunoblotted with anti-p27, anti-cyclin D1, and anti-cyclin D3 antibodies. Anti-β-actin was used as a loading control. **b**, **c**, **e**, **f** are representative of three separate experiments. The statistical analysis associated is available in the Supplementary Figure S2

As drug resistance commonly involves several mechanisms that are often closely interconnected with their genetic profile, for our next analysis we chose the MDA-MB-468 and BT-549 cells, as they show a “similar” genetic background (i.e., phosphatase and tensin homolog (*PTEN*), *RB1*, and *P53* mutations)^29^, which could help us to predict a “similar” sensitivity to TKIs^27^. We first evaluated whether the knockdown of Notch3 (siN3) or Notch1 (siN1) by small interfering RNA (siRNA) could affect cell growth or viability in such cells (Fig. 2a, d). Notably, both MDA-MB-468 (Fig. 2a) and BT-549 (Fig. 2d) cells display a more significant cell growth reduction after the selective depletion of Notch3 with respect to Notch1, measured by counting cell number until 6 days from the starting point, day 0 thus confirming previous data^14^. This effect could be due to the growth arrest of the cells, as the absence of Notch3 in both cell lines correlates with a significant upregulation of the cyclin-dependent kinase inhibitor p27^Kip1^ and downregulation of both the cyclins D1 and D3, known to be important protein regulators that exhibit dynamic changes during the cell cycle (Fig. 2b, e and Supplementary Figure S2a and c). Notably, the Notch1 silencing does not correlate with any significant changes of the same cell cycle regulators analyzed (Fig. 2c, f and Supplementary Figure S2b and d). These results demonstrate a specific role of Notch3 in the regulation of TNBC cell growth, as confirmed by the absence of viability of MDA-MB-468 clones stably deleted for *NOTCH3* (but not for *NOTCH1*), generated by using genome-editing CRISPR/Cas9 technique (data not shown).

**Fig. 3:**
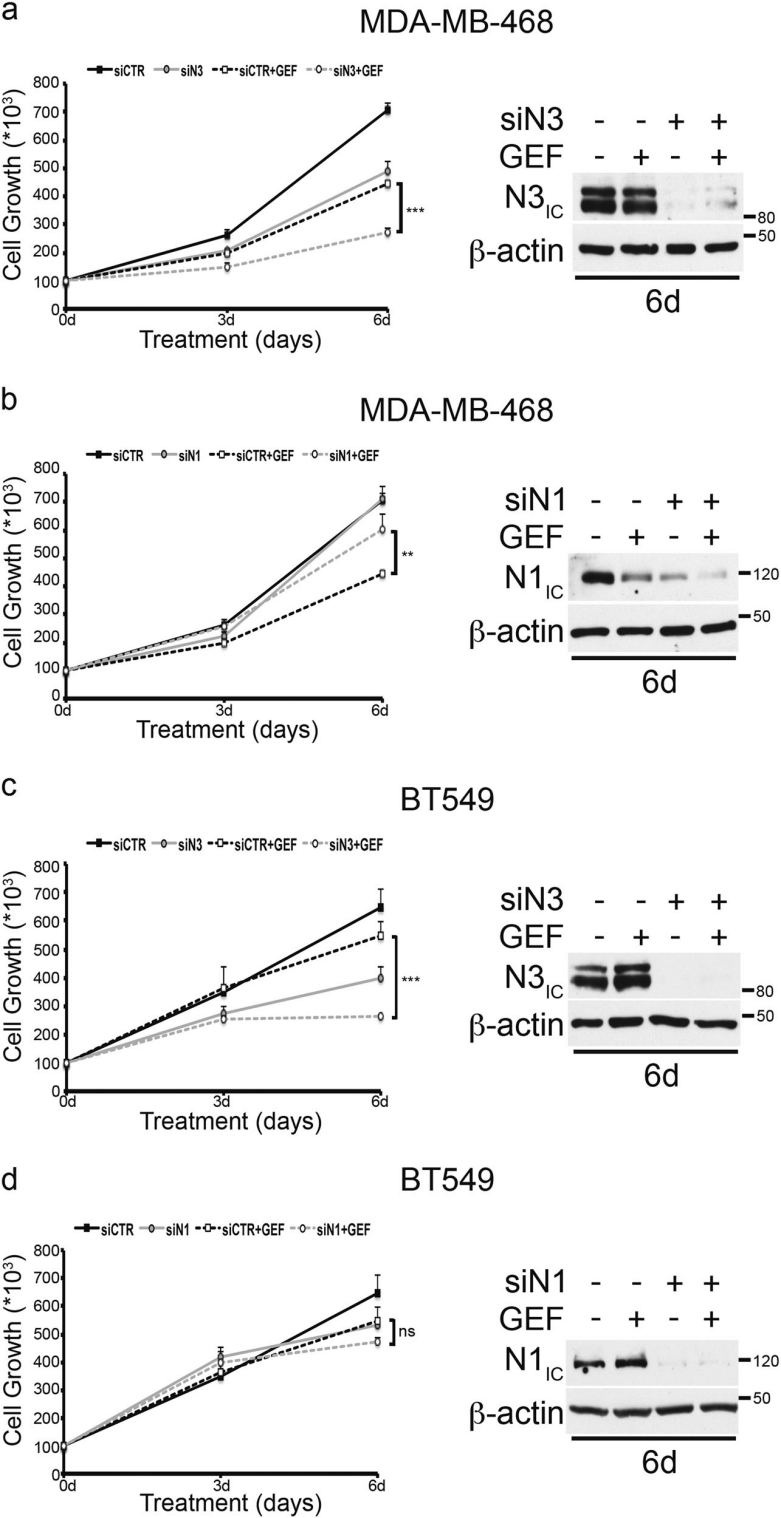
Notch3 downregulation (but not Notch1) sensitizes TNBC cells to TKI-gefitinib. **a**–**d** Left panels: inhibition of **a**, **b** MDA-MB-468 and **c**, **d** BT-549 cell growth was observed after gefitinib (GEF) treatment combined with Notch3 silencing in **a**, **c** but not with **b**, **d** Notch1 silencing. All the right panels showed in the figure represent western blotting of total extracts from cells described above against Notch3 (N3_IC_) and Notch1 (N1_IC_), to control the efficiency of the **a**, **c** Notch3 and **b**, **d** Notch1 silencing, respectively. Anti-β-actin was used as a loading control. All data are representative of at least three independent experiments, each in triplicate. Results shown in **a**, **b**, **c**, and **d** are expressed as the means average deviations and *P*-values were calculated using Student’s *T*-test (i.e., ns not significant; *P* > 0.05, *P ≤ 0.05, **P ≤ 0.01, ***P ≤ 0.001)

Previous studies suggested that selective Notch3 inhibition (rather than pan-Notch inhibition) combined with EGFR TKI therapy should be explored as a novel strategy in the treatment of lung cancer patients^21^. In keeping with these data, we observed that Notch3 silencing significantly enhances the gefinitib (GEF)-induced growth inhibition in both MDA-MB-468 and BT-549 cells (Fig. 3a, c, left panels: compare siCTR + GEF vs. siN3 + GEF), in a similar or even more extensive way observed after combined treatment with GSI plus TKI-GEF (Supplementary Figure S3a and b, left panels: compare siCTR + GEF vs. GSI + GEF). These data thus strongly suggests that Notch3 depletion rather than pan-Notch inhibitor is sufficient to sensitize TNBC to TKI-GEF (Fig. 3a vs. Supplementary Figure S3a; Fig. 3c vs. Supplementary Figure S3b, left panels: compare siN3 + GEF vs. GSI + GEF). The quality of Notch(s) silencing were monitored until 6 days by evaluating the expression of both Notch3 and Notch1 proteins (Fig. 3 and Supplementary Figure S3, all the right panels).

In addition, although Notch1 silencing does not induce any significant changes in BT-549 GEF-treated cells with respect to control cells (Fig. 3d, compare siCTR + GEF vs. siN1 + GEF), it seems to paradoxically increase the MDA-MB-468 cell growth in response to GEF (Fig. 3b, left panel: compare siCTR + GEF vs. siN1 + GEF). These data suggest a potential different role of the different Notch receptors expressed in the same TNBC context relative to the TKI-response, which remains to be fully elucidated.

### Dual targeting of EGFR and Notch3 increases both EGFR internalization and dephosphorylation, and decreases the EGFR nuclear localization

To understand how the Notch3-dependent TKI resensitization observed above could occur in TNBC cells, we initially examined whether EGFR turnover could be influenced by the absence of Notch3 rather than Notch1. To this purpose we focused our next studies on MDA-MB-468 cells, by evaluating both the EGFR subcellular localization and its tyrosine phosphorylation status, which is essential for EGFR to activate downstream mitogenic pathways and represents the basis for targeted therapy with TKIs^30^.

**Fig. 4:**
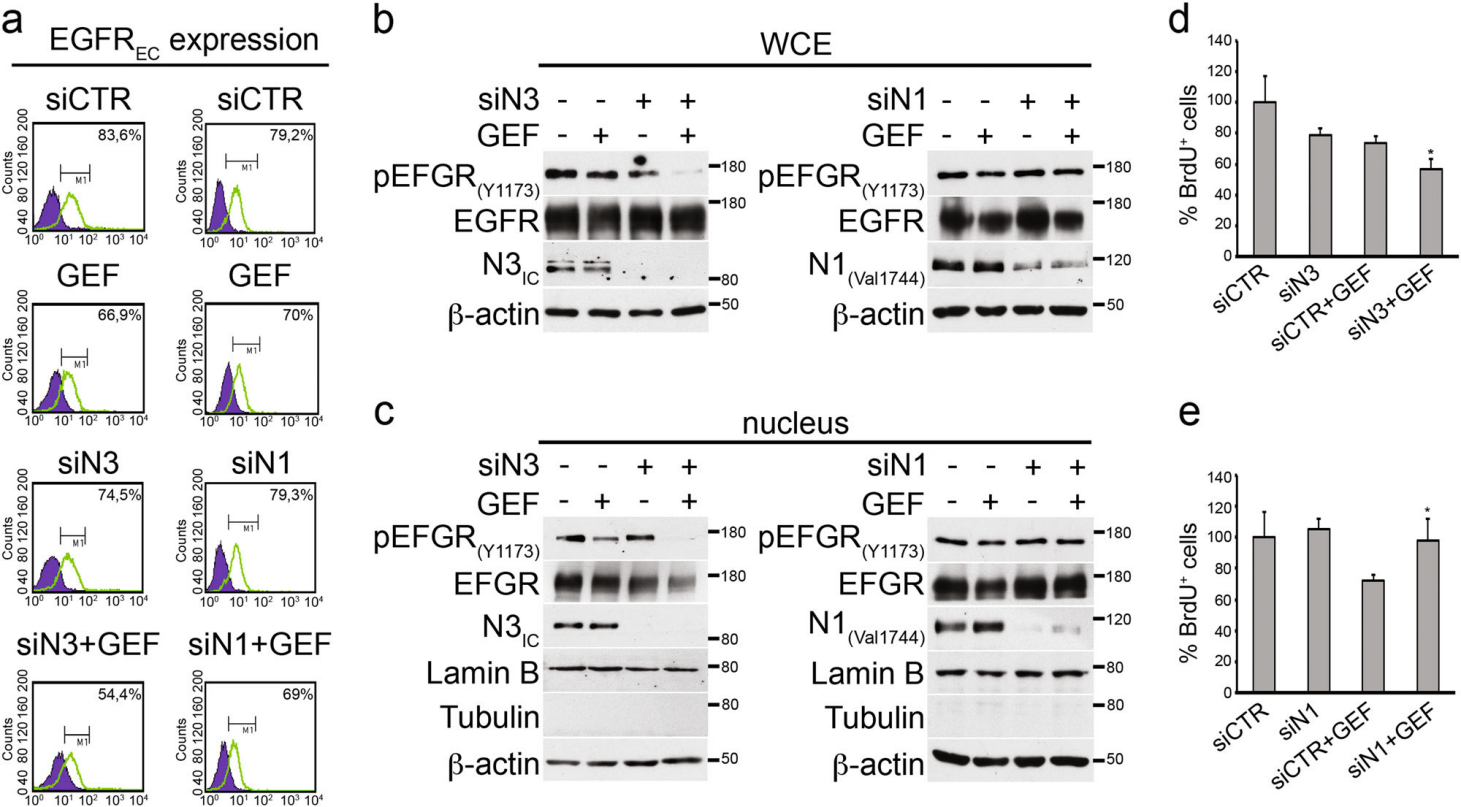
Combined Notch3 and EGFR targeting induces EGFR internalization but defect the nuclear-activated EGFR localization. **a** FACS analysis of the EGFR surface expression (EGFR_EC_) in MDA-MB-468 cells treated with gefitinib (GEF) alone or in combination with Notch3-silencing (siN3 + GEF) or Notch1 silencing (siN1 + GEF) for 6 days. **b** Whole cell extracts (WCE) and **c** nuclear extracts from the same cells used in **a** were immunoblotted with anti-EGFR and anti-pEGFR_(Y1173)_ antibodies, to evaluate the EGFR expression and phosphorylation, and with anti-N3_IC_ or anti-N1_Val1744_ antibody to control the efficiency of Notch3 (left panels) or Notch1 (right panels) silencing, respectively. Anti-lamin B and anti-tubulin were used as fraction markers; anti-β-actin was used as a loading control. **d**, **e** Proliferation analysis by BrdU assay (see Matherials and Methods section): compared with control cells (siCTR + GEF), the percentage of BrdU^+^ cells is lower after Notch3 silencing plus **d** GEF (siN3 + GEF) and not after Notch1 silencing plus **e** GEF (siN1 + GEF). All data are representative of at least three independent experiments, each in triplicate. Results are expressed as the means average deviations and *P*-values were calculated using Student’s *T*-test (i.e., *P ≤ 0.05)

The MDA-MB-468 cells were treated with GEF, alone or in combination with Notch3 or Notch1 silencing (siN3 + GEF or siN1 + GEF, respectively) for 6 days, followed by the analysis of the following: (1) the EGFR surface expression (EGFR_EC_) by fluorescence-activated cell sorting (FACS) analysis (Figs. 4a) and (2) the tyrosine-phosphorylated EGFR expression at 1173 residue (pEGFR_Y1173_) in both whole cell (Fig. 4b) and nuclear extracts (Fig. 4c). Notably, the absence of Notch3 amplifyies the GEF-dependent decrease of EGFR_EC_ surface-expressing cells (siN3 + GEF: 54,4% vs. GEF: 66,9%) (Fig. 4a, left panels) whereas Notch1 silencing does not (siN1 + GEF: 69% vs. GEF: 70%) (Fig. 4a, right panels). Similarly, Notch3 depletion leds to a significant decrease of pEGFR_Y1173_ expression, which appears rarely detectable in both total and nuclear extracts of GEF-treated Notch3-silenced cells (Fig. 4b, c, left panels, respectively). On the contrary, Notch1 silencing does not induce important alterations of pEGFR_Y1173_ expression neither in whole cell nor in nuclear extracts from GEF-treated cells (Figs. 4b, c, right panels, respectively). It has been reported that the full-length form of nuclear EGFR is involved in several mechanisms including cell proliferation^31^. Consistent with this, by measuring bromodeoxyuridine (BrdU)-positive cells during the combined experiments (after 4 days), we observed a significant decrease in the percentage of cells entering the S phase in GEF-treated Notch3-silenced cells with respect to GEF-treated cells (Fig. 4d, compare siCTR + GEF vs. siN3 + GEF). Notably, in keeping to what is shown above (Fig. 3b), we observed an increase in the percentage of proliferative GEF-treated Notch1-silenced cells with respect to their counterpart treated with GEF alone (Fig. 4e, compare siCTR + GEF vs. siN1 + GEF).

All these data suggest an important correlation between EGFR behavior and Notch3 receptor in TKI-response of TNBC cells, thus providing a rationale for a combined therapy approach with TKI-GEF and Notch3 inhibition.

### Rafts depletion correlates with EGFR dephosphorylation by PTPH1 phosphatase in TKI-resistant TNBC cells

In order to further understand the molecular mechanism underlying the EGFR-Notch3 crosstalk in TNBC, we investigated in more detail whether and how Notch3 may be involved in the regulation of the above described processes of EGFR subcellular localization and its phosphorylation/activation status.

**Fig. 5:**
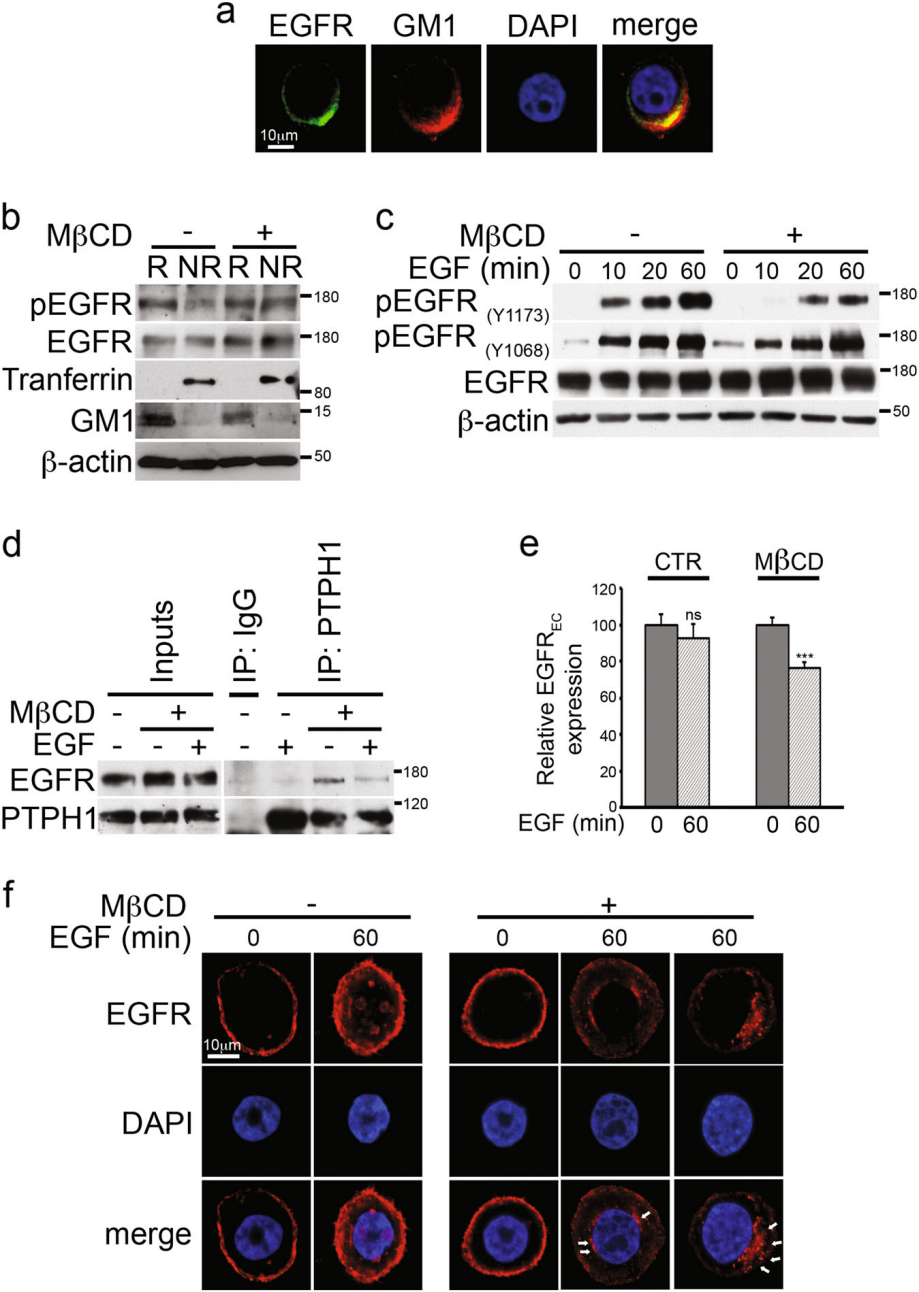
Rafts depletion induces endogenous EGFR-PTPH1 interaction, EGFR dephopshorylation, and its intracellular arrest in MDA-MB-468 TNBC cells. **a** Immunofluorescence assay (IF) was performed by using anti-EGFR (green) and anti-GM1 (red) antibodies to reveal the endogenous EGFR-rafts colocalization, shown in yellow (merge). Nuclei were DAPI labeled (blue). **b** Raft (R) and non-raft (NR) fractions derived from Methyl-β-cyclodextrin (MβCD)-treated and untreated cells were used for immunoblot assay with anti-pEGFR_(Y1173)_ (indicated as pEGFR) and anti-EGFR antibodies, to test activated and total EGFR expression in rafts compartment, respectively. Anti-transferrin and anti-GM1 antibodies were used as a fraction markers. **c** Cells have been activated with EGF ligand for the times indicated, in the presence or absence of MβCD: the expression of phospho-EGFR at tyrosine 1173 and 1068 residues and total EGFR was determined in whole cell extracts by immunoblot analysis using the specific indicated antibodies. **d**–**f** MDA-MB-468 cells were treated with MβCD and stimulated with EGF for 60 min: control or anti-PTPH1 antibody immunoprecipitates were probed with anti-EGFR, to detect the EGFR-PTPH1 binding, and with the anti-PTPH1 antibody, to show PTPH1 immunoprecipitated protein levels. The inputs indicated in the panel shows 5% of each total lysate **d**. Relative EGFR extracellular expression (EGFR_EC_) was evaluated by FACS **e**. IF assay was performed by using anti-EGFR (red) antibody to reveal the endogenous EGFR intracellular localization. Nuclei were DAPI labeled (blue). White arrows indicated peri-nuclear EGFR localization in EGF stimulated MβCD-treated cells (**f**). **a**, **f** Representative single plane confocal IF images captured using a × 60 oil objective. Scale bar: 10 μm. In both **b** and **c**, western blotting against the anti-β-actin was used as a loading control. All data are representative of at least three independent experiments, each in triplicate. Results shown in **e** are expressed as the means average deviations and *P*-values were calculated using Student’s *T*-test (i.e., ns, not significant *P* > 0.05, **P ≤ 0.01)

It has been shown that EGFR localizes within lipid rafts in different cell lines^32^ and this specific localization could induce different functional effects^33,34^. More recently, Irwin et al.^35^ have shown that EGFR localization to lipid rafts of TNBC cells may correlate with their resistance to EGFR TKI-induced growth inhibition. First, we confirmed the presence of EGFR within lipid rafts by using biochemical and confocal microscopy analyses: Fig. 5a shows that EGFR (green) strongly colocalizes with GM1 (red), a lipid raft glycosphingolipid specifically recognized by the Cholera toxin subunit B. Biochemical rafts isolation shown in the Fig. 5b confirms these data. Notably, the tyrosine-pEGFR expression, essential for its functional activity^36^ and predictive for target therapy efficiency with TKIs^30^ appears to be exclusive of raft compartment, as it moved to the non-rafts fractions in the presence of Methyl-β-cyclodextrin (MβCD), a drug which removes cholesterol from the plasma membrane, thus disrupting the integrity of membrane rafts microdomains (Fig. 5b). Interestingly, after MβCD treatment, we observe a clear defect in the increase of EGF-induced tyrosine phosphorylation of EGFR at 1173 residue (pEGFR_Y1173_) but not at 1068 residue (pEGFR_Y1068_) (Fig. 5c), thus suggesting the presence of potential different roles between EGFR phosphorylation pattern and function of different tyrosine phosphorylation sites^30^. These data indicate a possible relationship between rafts compartment integrity and EGFR/Y1173 dephosphorylation, which is known to have an important role in the therapeutic activity of EGFR TKI inhibition through the involvement of the tyrosine phosphatase H1 (PTPH1)^37^.

Several protein tyrosine phosphatases (PTPs) dephosphorylate EGFR at Y1173 (alone or together with other residues)^38,39^. Among them, the PTPH1 specifically catalyzes EGFR/Y1173 dephosphorylation (and not EGFR/Y1068 dephosphorylation), thus finally increasing non-TNBC BC sensitivity to TKIs, including GEF^37^. The EGFR/PTPH1 direct interaction is closely required to favor the therapeutic targeting of EGFR itself^37^. In agreement with this, we observed that MβCD-treated TNBC cells showed high levels of endogenous EGFR-PTPH1 interaction (Fig. 5d), thus suggesting the possible PTPH1 involvement in the observed decreased levels of EGFR/Y1173 phosphorylation after rafts depletion (Fig. 5c). Interestingly, the EGFR-PTPH1 interaction disappears after MβCD plus EGF ligand (Fig. 5d), probably due to the EGF-dependent endocytic events of ligand-activated EGFRs which may influence the kinetics of EGFR availability to PTPs-mediated dephosphorylation^40^. In keeping with this, we observed a decreased extracellular EGFR expression (EGFR_EC_) in MβCD-treated cells with respect to untreated cells, upon stimulation with EGF (Fig. 5e), despite the natural slowdown of EGFR endocytic trafficking in MDA-MB-468 cells due to their known saturated endocytic machinery^41^. In agreement with previous data^42^, our results suggest that rafts depletion may allow the internalization of ligand-occupied EGFR. Following ligand binding and receptor phosphorylation/activation, pEGFR is endocytosed and commonly transported to lysosome where it is degraded^43^. In our experiments, we do not observe decreased levels of total EGFR expression after rafts depletion (Fig. 5c, d), thus suggesting that removal of EGFR from the cell surface observed in MβCD-treated cells may be correlated to a different mechanism of EGFR downregulation, not involving receptor degradation. Since it has been demonstrated that many tumor cells which overexpress EGFR, including the MDA-MB-468 cells, have limited ligand-stimulated EGFR degradation^44^ and that tyrosine dephosphorylation of EGFR is correlated with an increased EGFR stability^37^, we wanted to know where the EGFR accumulated after MβCD treatment, in order to completely understand how signaling by the EGFR is terminated. To this purpose, cells were treated with or without MβCD and stimulated with EGF ligand for 60 min, followed by the immunostaining with anti-EGFR antibody (Fig. 5f): confocal analysis shows that rafts depletion correlates with the accumulation of EGFR at a peri-nuclear level (white arrows) whereas the majority of MβCD-untreated cells (EGF stimulated) show spots of nuclear EGFR, which represents a specific localization known to be associated with resistance to EGFR-targeted therapies^31^. In addition, the same control cells stimulated with EGF ligand show persistent high levels of EGFR cell surface expression (Fig. 5f), thus confirming the saturation of the endocytic machinery previously mentioned^41^.

Together, these data indicate that EGFR trafficking is retained outside the nucleus in MDA-MB-468 TNBC cells in response to the rafts-disrupting agent, MβCD.

### Notch3 inhibition by siRNA mimics rafts depletion effects on EGFR in TKI-resistant TNBC cells

We have previously shown that Notch3 receptor constitutively localizes to lipid rafts of Notch3 overexpressing lymphocytes, thus contributing to sustain the signaling pathways responsible of the T-cell leukemia development^45^. Here we first hypothesized that both Notch3 and EGFR receptors could share the same localization to directly interact, leading to the observed EGFR-TKI resistance process in TNBC cells. Surprisingly, both confocal analysis (Supplementary Figure S4a, upper panels) and biochemical rafts isolation with or without MβCD treatment (Supplementary Figure S4b) show that in MDA-MB-468 cells Notch3 receptor (N3_EC_) is widely expressed in all the cell surface, whereas Notch1 receptor appears to be restricted to lipid rafts microdomains (Supplementary Figure S4a, lower panels, and S4b). Thus, Notch3 and EGFR do not completely colocalize (Supplementary Figure S4c, upper panels), whereas Notch1 shows a strong rafts colocalization with EGFR (Supplementary Figure S4c, lower panels). Notably, by in situ proximity ligation assay (PLA), we still observed the endogenous Notch3/EGFR complex all around the cell membrane (Supplementary Figure S4d) while Notch1/EGFR complex seems to be mainly restricted to a limited portion of the membrane (Supplementary Figure S4e), reflecting their strictly shared localization (Supplementary Figure S4c, lower panels).

**Fig. 6:**
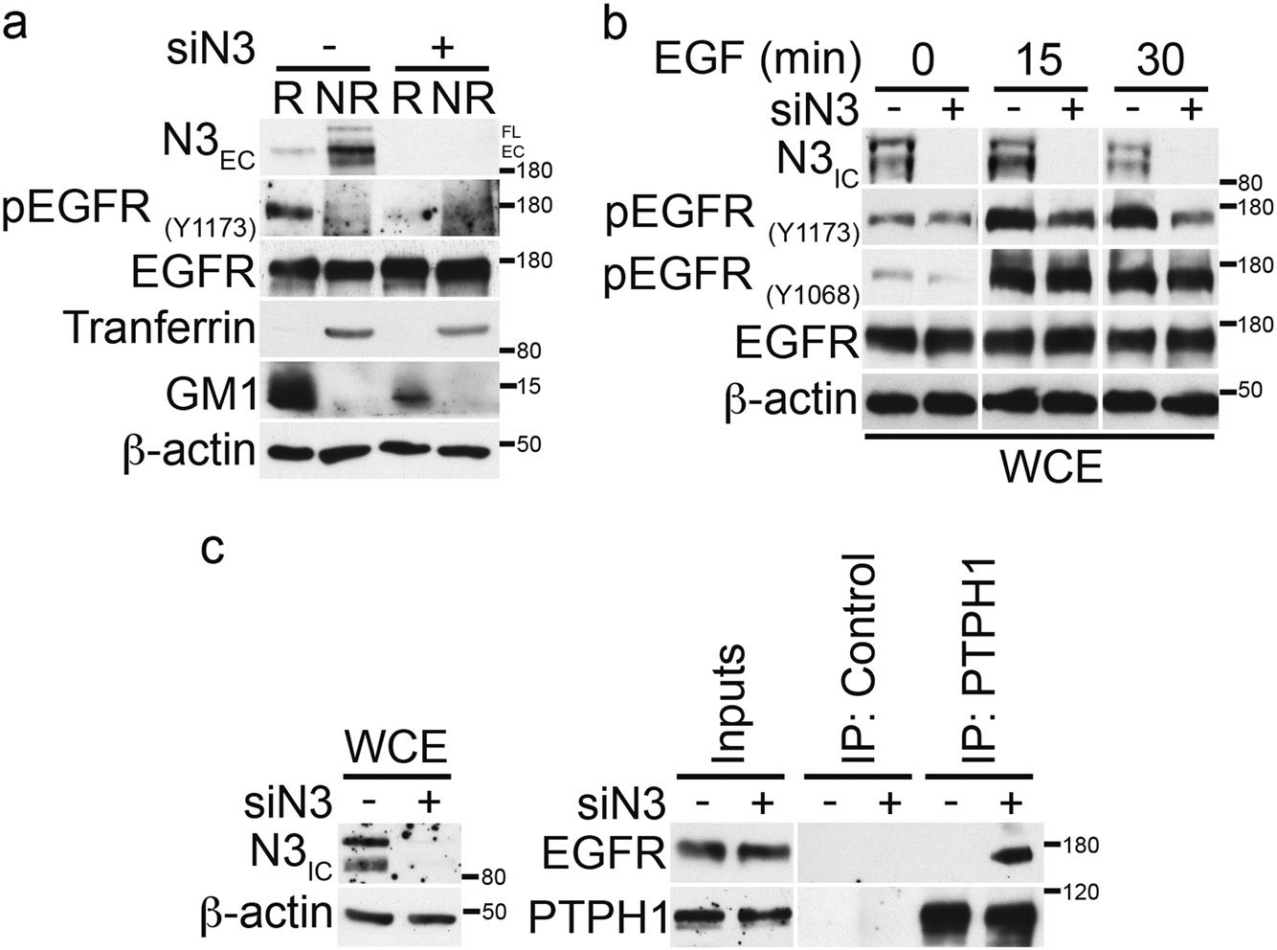
Notch3 downregulation induces EGFR dephosphorylation by promoting the endogenous EGFR/PTPH1 interaction. **a** Raft (R) and non-raft (NR) fractions derived from 6 days of Notch3-silenced cells were used for immunoblot assay with anti-N3_EC_, anti-pEGFR_(Y1173)_, and anti-EGFR antibodies, to test the effect of Notch3 downmodulation on EGFR-rafts localization. Anti-transferrin and anti-GM1 were used as a fraction markers. **b** Cells have been activated with EGF ligand for the times indicated, combined or not with Notch3 silencing for 3 days: the expression of phospho-EGFR at tyrosine 1173 and 1068 residues and total EGFR was determined by immunoblot analysis using the specific indicated antibodies. **c** Control or anti-PTPH1 antibody immunoprecipitates from control and Notch3-silenced cells were probes with anti-EGFR, to detect the EGFR-PTPH1 binding, and with the anti-PTPH1 antibody, to show PTPH1 immunoprecipitated protein levels. The inputs indicated in the panel shows 5% of each total lysate (right panels). Whole cell extracts (WCE) were incubated with anti-N3_IC_ antibody to control the efficiency of Notch3 silencing (left panels). In all panels **a**, **b** and **c**, western blotting against the anti-β-actin was used as a loading control. The results are representative of three independent experiments

These results suggest the existence of a different relationship between Notch3 or Notch1 and EGFR in TNBC. However, in order to deepen inside the molecular mechanism related to the TKI-GEF resensitization of TNBC cells observed only when Notch3 (and not Notch1) is depleted (Fig. 3), here we further investigated how the EGFR-rafts localization could be influenced when Notch3 is depleted. Similar to what happens in MβCD-treated cells (Fig. 5b-d), in the absence of Notch3 the tyrosine phosphorylation of EGFR at 1173 residue (pEGFR_Y1173_) disappears and this event does not involve receptor degradation, as EGFR total levels remain unchanged (Fig. 6a). Notably, after Notch3 depletion, we observed a clear defect in the increasing levels of EGF-induced tyrosine phosphorylation of EGFR at 1173 residue (pEGFR_Y1173_) but not at 1068 residue (pEGFR_Y1068_) (Fig. 6b), as already shown after rafts depletion (Fig. 5c). For this reason, we further investigated whether Notch3 could influence the EGFR/Y1173 dephosphorylation by the phosphatase PTPH1, by using co-immunoprecipitation assay. In agreement with the above results (Fig. 5d), we observed that the absence of Notch3 is able to induce the endogenous EGFR/PTPH1 interaction (Fig. 6c), thus suggesting a possible link between Notch3, EGFR-rafts localization and EGFR dephosphorylation event by PTPH1.

**Fig. 7:**
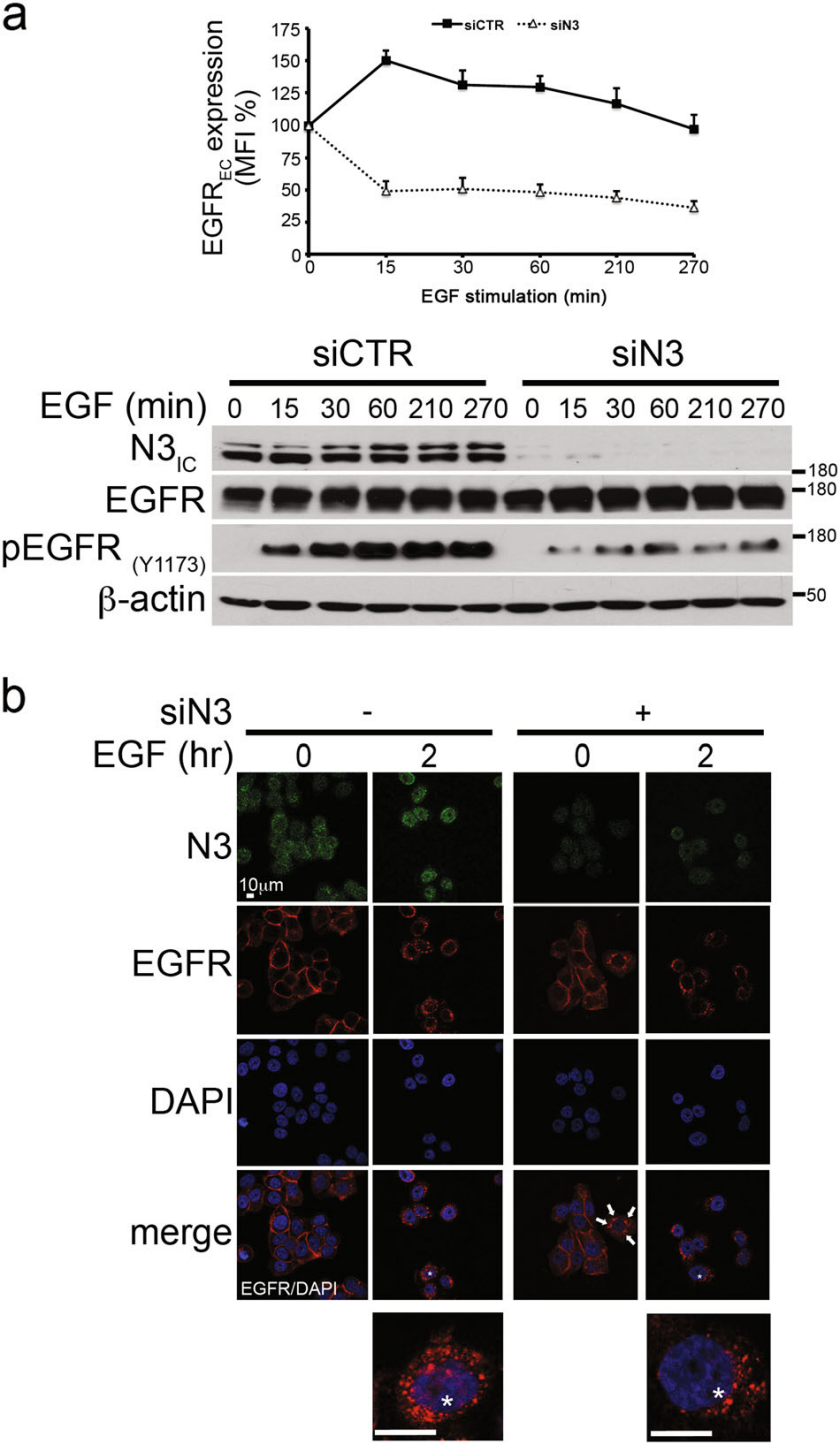
Notch3 downregulation induces EGFR internalization and intracellular arrest. **a** Upper panel: FACS analysis of the EGFR surface expression (EGFR_EC_) in control (siCTR) and Notch3-silenced (siN3) cells after EGF stimulation for the time indicated, shown as percentage of the mean fluorescence intensity (MFI) respect to the EGF-untreated cells (*t* = 0). lower panel: Western blot analysis of the total extracts from the same cells probed with anti-Notch3 (N3_IC_) antibody, to test the efficiency of Notch3 silencing, and with anti-EGFR and anti-pEGFR_(Y1173)_ antibodies, to evaluate the EGFR expression. The β-actin expression was used as loading control. **b** MDA-MB-468 cells were Notch3-silenced for 48 h and EGF-treated for 2 h: Immunofluorescence assay (IF) was performed by using anti-Notch3 green) or anti-EGFR (red) antibodies to test the efficacy of Notch3 silencing and to reveal the endogenous EGFR intracellular localization, respectively. Nuclei were DAPI labeled (blue). EGFR/DAPI merge is shown. White arrows indicate peri-nuclear EGFR localization in Notch3-silenced cells. The * indicate the higher magnification of a single EGF-stimulated control cell (left) and Notch3-silenced (right) cell. All the panels are representative single plane confocal IF images captured using a × 60 oil objective. Scale bar: 10 μm. The results are representative of three independent experiments

In addition, we also observed that the absence of Notch3 correlates with a rapid and persistent EGFR downregulation from the cell surface, as revealed by the decrease of EGFR_EC_ mean fluorescence intensity (MFI) in Notch3-depleted cells (siN3) with respect to control cells (siCTR) after EGF stimulation (Fig. 7a, upper panel). As expected, treatment of MDA-MB-468 cells with EGF until 270 min results in an increased EGFR surface expression (Fig. 7a, upper panel), also supported by the unchanged levels of total EGFR protein (Fig. 7a, lower panels), as previously reported (Fig. 5f and^41^). Interestingly, despite the increased EGFR internalization observed in the absence of Notch3, although the pEGFR_Y1173_ expression decreases, the EGFR total levels does not change, thus suggesting that Notch3 depletion (such as rafts depletion) could correlate with an increased dephopshorylated EGFR endocytosis followed by its intracellular shuttling blockade rather than sorting for intracellular degradation. Using immunofluorescence staining, we obtained additional evidence in support of the Notch3-depletion dependence of EGFR intracellular fate. As shown in the Fig. 7b, after 2 h of EGF stimulation combined with Notch3 silencing, we observed that EGFR localizes preferentially at a peri-nuclear level, similarly to what observed after MβCD treatment (Fig. 5f). Interestingly, a few cells show a similar EGFR staining also without EGF stimulation (Fig. 7b, see white arrows), thus suggesting that Notch3, alone, may influence the EGFR internalization also through ligand-independent mechanisms (data not shown).

Together these results demonstrate that Notch3 depletion mimics the effects of rafts depletion on EGFR, as the Notch3 silencing correlates with EGFR dephosphorylation (by PTPH1) and its persistent internalization, followed by intracellular arrest.

## Discussion

Among the EGFR TKIs, GEF and erlotinib were the first to be approved by Food and Drug Administration for treatment of NSCLC^46^. These drugs inhibit the EGFR kinase activity, finally resulting in proliferation inhibition, cell cycle progression delay, and apoptosis^47^. Although EGFR TKIs show good response rates and progression free survival in NSCLC patients with *EGFR* gene mutations, acquired resistance to TKIs therapy is commonly reported, often due to multiple mechanisms including *EGFR* additional mutations^48,49^, activation of redundant kinase signaling pathway, or EGFR downstream molecules^2^. As activating mutations of *EGFR* in BC are rare, it is uncertain whether some of the above mentioned mechanisms observed in NSCLC are involved in the failure of clinical trials with TKIs in TNBC. One possible explanation for the lack of response to current targeted therapies is that most TNBCs are not exclusively dependent on EGFR signaling for their survival but involve the activation of alternative receptors and pathways. As some NSCLC patients with wild-type *EGFR* gene amplification and wild-type *KRAS* also respond to EGFR TKIs^50^, it may be that these alternative resistant pathways need to be blocked in wild-type EGFR overexpressing TNBC patients to increase TKIs therapy efficacy.

Here we demonstrate that Notch3 (but not Notch1) is strongly involved in the TNBC resistance to TKIs, as Notch3 depletion induces the resensitization of TNBC cells to GEF treatment. These results indicate that Notch3 specifically functions in these cells without invoking contributions from other Notch receptors, thus supporting the importance of a selective Notch3 therapeutic targeting in order to avoid the known toxicity associated with pan-Notch inhibition^51^. The significant correlation observed between Notch3 and EGFR in a large group of human TNBC patients supports these data.

Mechanistically, we show that Notch3 depletion induces the downregulation of EGFR cell surface expresssion and function by promoting its dephosphorylation via PTPH1 and its intracellular arrest, similar to what observed after rafts-disrupting treatments. Interestingly, we have shown that MDA-MB-468 TNBC-resistant cells show a strong lipid rafts localization of the activated EGFR. In addition, it has been demonstrated that EGFR overexpression correlates with the natural saturation of its endocytic trafficking^41^. Consequently, in these cells EGFR seems not to be available to be downregulated, thus finally retaining a constitutive higher surface expression, known to be associated with cell growth^44^. In this scenario, the Notch3 silencing is able to favor a hypothetical shift of the EGFR from rafts to non-rafts compartment, thus moving it to a membrane localization, which may be available to subsequent downregulating events. Possible mechanisms able to attenuate EGFR signaling include dephosphorylation of the EGFR, removing it from the cell surface and allowing degradation of the receptor or sequestration into intraluminal vesicles^52^. PTPH1 is a phosphatase able to specifically dephosphorylate EGFR at tyrosine Y1173 residue^53^, thereby regulating EGFR interaction with ER and the subsequent ER^+^ BC sensitivity to TKIs treatment^37^. In both Notch3-silenced and MβCD-treated cells, we observed a strong EGFR-PTPH1 endogenous interaction which is correlated with a significant decrease of pEGFR_Y1173_ expression, thus suggesting that EGFR dephosphorylation by PTPH1 may represent an important event of the observed Notch3-dependent increased response to TKI-GEF. Further studies are required to understand if PTPH1 could be recruited into lipid rafts before EGFR moving, as PTPH1 is involved in the non-clathrin endocytosis of EGFR in lung cancer^54^ and/or if the EGFR internalization is required for the PTPH1-EGFR interaction, as occurs for other tyrosine phosphatases function on EGFR itself^55^.

EGFR dowregulation commonly involves a clathrin-mediated endocytosis event dependent on physiological concentrations of growth factors^42^. Moreover, it has been demonstrated that EGFR internalization occurs also under various stress conditions (such as treatment with drugs) through the involvement of p38 mitogen-activated protein kinase (MAPK), finally leading to its arrest in endosomes, without recycling^56^. Here we demonstrate that Notch3 depletion induces an increased EGFR internalization, more evident in combination with GEF, thus leading to the EGFR intracellular accumulation and not degradation. Interestingly, Notch3 is able to positively control the levels of MAPK phosphatase 1 (MKP-1), thus decreasing the levels of phosphorylated p38, a canonical MKP-1 target^57^. These data suggest that the absence of Notch3 may mimic a stress condition able to activate p38 and favor the EGFR non-canonical internalization. Moreover, it has been also shown a direct crosstalk between PTPH1 and p38 MAPK in promoting Ras oncogenesis and regulating stress response: in particular, PTPH1 represents a p38γ-specific phosphatase^58^ and PTPH1 phosphorylation by p38 is required to favor the PTPH1 dephosphorylation activity on its targets, such as EGFR/Y1173^53^. These observations further support our hypothesis of an important involvement of Notch3-PTPH1 axis in the regulation of EGFR internalization machinery in TNBC. Moreover, the EGFR intracellular accumulation observed in Notch3-depleted cells correlated with a defect in its nuclear localization, further suggesting that removal of EGFR from the cell surface may help to evade survival signaling and enhances drug-induced cell death, in accordance with a previous report^42^.

Collectively, our data suggest an important role of Notch3 in regulating the EGFR subcellular localization and function in TNBC cells, thus contributing as an intrinsic resistant factor to anti-EGFR therapies, whose failure is often dependent on different EGFR subcellular localization that elicit distinctly different and also overlapping signals^35,59^ and can make the receptor unavailable to be targeted.

Due to the heterogeneity of the TNBC and its poor outcome, subtyping through robust predictive and prognostic biomarkers that may contribute to therapy resistance is crucial for understanding the molecular mechanism underlying EGFR inhibitors sensitivity and further discuss the possible perspective on anti-EGFR therapies in TNBCs. In this view, here we observed an overlapped TKI-response in MDA-MB-468 and BT-549 cell lines, which both express a constitutive EGFR activation in a *PTEN*-null background, that is a known common combination of aggressive and drug-resistant subset of TNBC^60^. Based on our results, we can suggest Notch3 as one driver of an oncogenic signaling network, which may influence this intrinsic EGFR-TKI drug resistance in TNBC cells with such a similar molecular signature, finally allowing the design of specific target therapy protocols, which may include Notch3 inhibition as a potential approach for overcoming TKIs resistance.

## Materials and methods

### Cell culture and treatments

Human BC MDA-MB-468, MDA-MB-231, HCC1143, and BT-549 TNBC cell lines were obtained from ATCC; HCC38, BT-20, HS578T, and MDA-MB-453 TNBC cell lines were kindly provided by Professor JV Olsen (Novo Nordisk Foundation Center for Protein Research, University of Copenhagen, Denmark). All TNBC cells were maintained in accordance with the ATCC’s instructions and all are mycoplasma free.

Cell viability was measured by the Trypan blue dye exclusion assay (Sigma-Aldrich, St Louis, MO, USA, Catalog number T8154). Cells were treated with the following compounds: 5 mM MβCD (Sigma-Aldrich, Catalog number C4555); 3 μM GEF (Iressa, Selleckem, Houston, TX, USA; Catalog number ZD1839), 100 nM EGF Ligand (EGF; Gibco, Life Technologies, Carlsbad, CA, USA; Catalog number PHG0315); 10 μM of GSI IX (DAPT) (Calbiochem, Darmstadt, Germany; Catalog number 565770).

### siRNA silencing

Cell were transfected as previously described^61^ with siRNAs anti-Notch3 (Catalog number sc-37135), Notch1 (Catalog number sc-36095), and corresponding control scrambled siRNAs (Catalog number sc-37007), all from Santa Cruz Biotechnology (Santa Cruz, Dallas, TX, USA).

### Protein extract preparation, immunoprecipitation, and immunoblot analysis

Protein extract preparation^62^, immunoprecipitation assay^63^, immunoblotting assays^64^, and sucrose gradient for rafts isolation^45^ were performed as described elsewhere. Primary antibodies were as follows: anti-Notch3 (Catalog number 2889), anti-Notch1 (Catalog number 2421), anti-activated Notch1 (N1_Val1744_), anti-EGFR D38B1 (Catalog number 4267S), anti-phospho-EGFR Y1173 (53A5-Catalog number 4407S), and anti-phospho-EGFR Y1068 (Catalog number 2234S), all from Cell Signaling (Danvers, MA, USA); anti-α-tubulin (Catalog number sc-8035), anti-Lamin B M20 (Catalog number sc-6217), anti-p27 C19 (Catalog number sc-528), anti-cyclin D1 M20 (Catalog number sc-718), anti-cyclin D3 C16 (Catalog number sc-182), anti-Notch1 L18 (N1_EC_) (Catalog number sc-23299), anti-transferrin H65 (Catalog number sc-21011), and anti-PTPH1 (Catalog number sc-515181), all from Santa Cruz Biotechnology; anti-β-actin (Catalog number A5441) and anti-cholera toxin B subunit peroxidase conjugate (GM1, Catalog number C3741) from Sigma-Aldrich; and anti-Notch3 5E1 (N3_EC_) antibody was kindly provided by Professor A Joutel^65^.

### Immunofluoresce assay and confocal imaging

Immunofluorescence staining and in situ PLA were performed as described elsewhere^66^. Primary antibodies were as follows: rabbit anti-Notch3 M-134 (Catalog number sc-5593), mouse anti-EGFR 528 (Catalog number sc-120), and rabbit anti-Notch1 L18 (Catalog number sc-23299) from Santa Cruz Biotechnology; anti-GM1 (Life Technologies; 595-Cy3 conjugated, Catalog number C34777); mouse anti-Notch3EC, Clone 1E4 (Millipore, Billerica, MA, USA; Catalog number MABC594); and rabbit anti-EGFR (Proteintech, Rosemont, IL, USA; N-Terminal, Catalog number 22542–1-AP). Secondary antibodies were as follows: Alexa Fluor 594- and 488- conjugated, respectively, both anti-mouse and anti-rabbit (Molecular Probes, Life Technologies). Nuclei were counterstained with Hoechst reagent. Single, plane confocal images in the center of the cell were acquired using an inverted Olympus iX73 microscope equipped with an X-light Nipkow spinning-disk head (Crest Optics, Rome, Italy) and Lumencor Spectra × Led illumination. Images were collected using a CoolSNAP MYO CCD camera (Photometrics, Tucson, AZ, USA) and MetaMorph Software (Molecular Device, Sunnyvale, CA, USA) with a × 60 oil objective.

### Immunohistochemistry

Studies on human samples (already obtained for diagnostic purposes) were performed according to the standards of the local ethical committee. Immunoistochemistry was performed as previously described^11^, by using the following antibodies: anti-Notch1 C-20R (Santa Cruz Biotechnology; Catalog number sc-6014, 1:50 dilution), anti-Notch3 M-134 (Santa Cruz Biotechnology; Catalog number sc-5593, 1:50 dilution), and anti-EGFR D38B1 (Cell Signaling; Catalog number 4267S, 1:50 dilution). The sample was defined as negative when the number of stained cells was < 1% of the tumor cell population. The percentage of positive cells for each marker analyzed is reported in the Figure.

### In vitro BrdU assay and FACS analysis

In vitro BrdU assay was performed as described elsewhere^67^. For EGFR_EC_ expression, cells were stained with anti-EGFR antibody (Santa Cruz Biotecnology; Catalog number sc-528) or normal mouse IgG (Santa Cruz Biotecnology; Catalog number sc-2025) used as a negative control. Data were analyzed on a FACS-Calibur with CellQuest software (BD Bioscience, San Jose, CA, USA), as previosuly described^68^.

### In silico analysis of TNBC patients’ deposited data

Tumor samples from a cohort of 198 TNBC patients (GEO ID: GSE76124) and 579 TNBC patients (GEO ID: GSE31519) were selected and analyzed for the correlation between *NOTCH1* or *NOTCH3* and *EGFR* genes. The expression values were filtered in each analysis utilizing the expression probe set 218902_at representing *NOTCH1*, 203238_s_at representing *NOTCH3*, and 201983_s_at representing *EGFR*. The expression value of *NOTCH1*, *NOTCH3*, and *EGFR* for both datasets was in log2 scale after normalization of the data with the RMA algorithm. The index Pearson’s *r* coefficient correlation and reported *P*-values were calculated using GraphPad Prism software Version 6.0 (La Jolla, CA, USA) for both datasets. Plots were generated and data were converted to standard score for the plots using GraphPad Prism Version 6.0.

### Statistical analysis

All results were reported as the mean ± SD of at least three independent experiments, each performed in triplicate. Student’s *t*-test for unpaired samples was used to assess differences among groups, with similar variance. A *P*-value of 0.05 was considered statistically significant (NS *P* > 0.05; *P ≤ 0.05; **P ≤ 0.01; ***P ≤ 0.001). We estimated the sample size considering the variation and mean of the samples. No statistical method was used to predetermine sample size. No samples were excluded from any analysis.

## Electronic supplementary material

Supplementary Information

Supplementary Figure Legends

Supplementary Figure S1

Supplementary Figure S2

Supplementary Figure S3

Supplementary Figure S4
