# Supplementary material for: NOTCH3 inactivation increases triple negative breast cancer sensitivity to gefitinib by promoting EGFR tyrosine dephosphorylation and its intracellular arrest
Source: Oncogenesis. 2018 May 25;7(5):42. doi: 10.1038/s41389-018-0051-9 (PMC5968025; doi:10.1038/s41389-018-0051-9)
Supplement: Supplementary file 1 — Supplementary Information [file 41389_2018_51_MOESM1_ESM.doc]

In the Supplementary Information there are included 5 files, as follow:

- Supplementary Figure S1 (.jpg)

- Supplementary Figure S2 (.jpg)

- Supplementary Figure S3 (.jpg)

- Supplementary Figure S4 (.jpg)

- Supplementary Figures Legends (.docx)
