# Supplementary material for: NOTCH3 inactivation increases triple negative breast cancer sensitivity to gefitinib by promoting EGFR tyrosine dephosphorylation and its intracellular arrest
Source: Oncogenesis. 2018 May 25;7(5):42. doi: 10.1038/s41389-018-0051-9 (PMC5968025; doi:10.1038/s41389-018-0051-9)
Supplement: Supplementary file 2 — Supplementary Figure Legends [file 41389_2018_51_MOESM2_ESM.doc]

**Supplementary Figure S1. TNBC screening for EGFR, Notch3 and Notch1 protein expression.** (a) Western blot analysis of EGFR, Notch3 and Notch1 proteins expression on a subset of TNBC selected cells (MDA-MB-468; HCC1143; HCC38; BT-20; HS578T; MDA-MB-453; BT-549; MDA-MB-231) compared to MCF10A cell line used as normal breast sample.

**Supplementary Figure S2. Notch3 silencing (rather than Notch1) influences the TNBC cell growth.** In all panels (a), (b), (c), (d) is shown the relative Optical densitometry (OD) of N3_IC_ (a,c) or N1_IC_ (b,d), p27, cyclin D1 (D1) and cyclin D3 (D3) protein expression levels represented in the Figure 2b, 2c (for MDA-MB-468 cells) and 2e, 2f (for BT-549 cells). Results are shown as means average deviations of three separate Notch silencing experiments and P-values were calculated using Student’s T-test (i.e., ns, not significant P>0,05; * P≤0.05; **P≤0.01; ***P≤0.001).

**Supplementary Figure S3. Effects of combined GSI IX (DAPT) plus GEF treatment on TNBC cells.** (a,b) Left panels: analysis of MDA-MB-468 (a) and BT-549 (b) cell growth after 0-3-6 days of gefitinib (GEF) treatment combined with γ-secretase inhibition (GSI+GEF) in MDA-MB-468 (a) and BT-549 (b) cells. Right panels represent western blot of total extracts from the same cells in (a) and (b) at 6 days, against Notch3 (N3_IC_) and activated-Notch1 (N1_Val1744_), to control the efficiency of the GSI treatment. Anti-β-actin was used as a loading control. All data are representative of at least three independent experiments, each in triplicate. Results shown in panels (a) and (b) are expressed as the means average deviations and P-values were calculated using Student’s T-test (i.e., *P≤0.05; **P≤0.01).

**Supplementary Figure S4. Notch3 and Notch1 membrane localization in MDA-MB-468 TNBC TKI-resistant cells.** (a) Immunofluorescence assay (IF) was performed by using anti-Notch3 (grenn, upper panel) or anti-Notch1 (green, lower panel) and anti-GM1 (red) antibodies to reveal the endogenous Notch(s) receptor-rafts colocalization, shown in yellow (merge). Nuclei were DAPI labeled (blue). (b) Raft (R) and Non-Raft (NR) fractions derived from Methyl-βcyclodextrin (MβCD)-treated and untreated cells were used for immunoblot assay with anti-N3_EC_ and anti-N1_EC_ antibodies, to test Notch(s) expression in rafts compartment. Anti-transferrin and anti-GM1 antibodies were used as a fraction markers; anti-β-actin was used as a loading control. (c) IF assay was performed by using anti-Notch3 (green, upper panel) or anti-Notch1 (green, lower panel) and anti-EGFR (red) antibodies to reveal the endogenous Notch(s) receptor-EGFR colocalization, shown in yellow (merge). Nuclei were DAPI labeled (blue). (d,e) Endogenous Notch3/EGFR (d) and Notch1/EGFR (e) interaction analyzed by *in situ* proximity ligation assay (PLA), detecting single interaction pairs of native proteins displayed as red signal by confocal microscopy. Negative controls lacking one of the primary antobody (only Notch3 (d); only Notch1 (e); only EGFR (d,e)). Protein complexes were visualized in red and indicated with white arrows; nuclei were DAPI labeled (blue).

All the panels (a), (c) and (d) are representative single plane confocal IF images captured using a 60X oil objective. Scale bar: 10 μm. The results are representative of three independent experiments.
