# Supplementary figures and images for: NOTCH3 inactivation increases triple negative breast cancer sensitivity to gefitinib by promoting EGFR tyrosine dephosphorylation and its intracellular arrest
Source: Oncogenesis. 2018 May 25;7(5):42. doi: 10.1038/s41389-018-0051-9 (PMC5968025; doi:10.1038/s41389-018-0051-9)

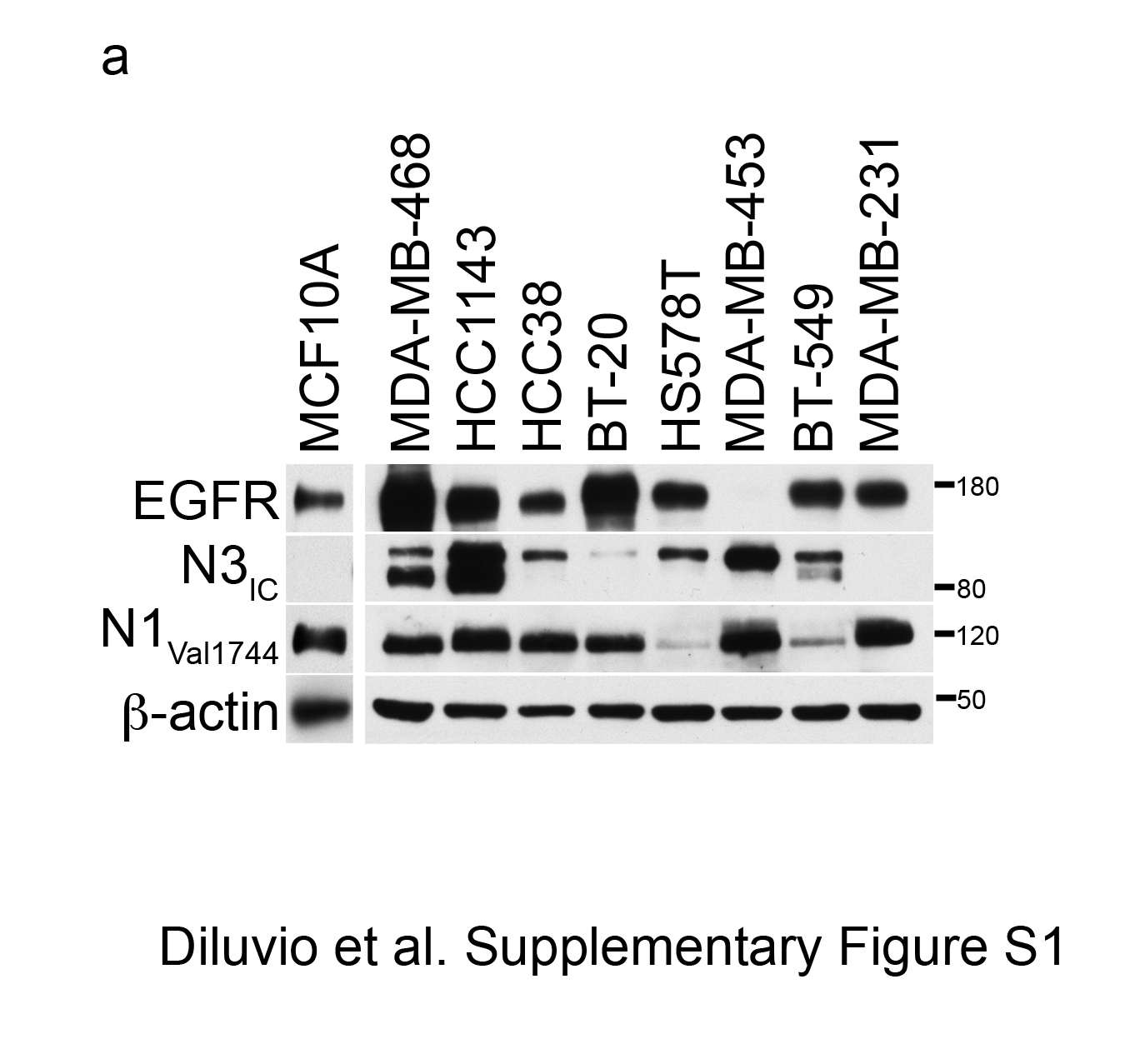

Supplement: Supplementary file 3 — Supplementary Figure S1 [file 41389_2018_51_MOESM3_ESM.jpg]

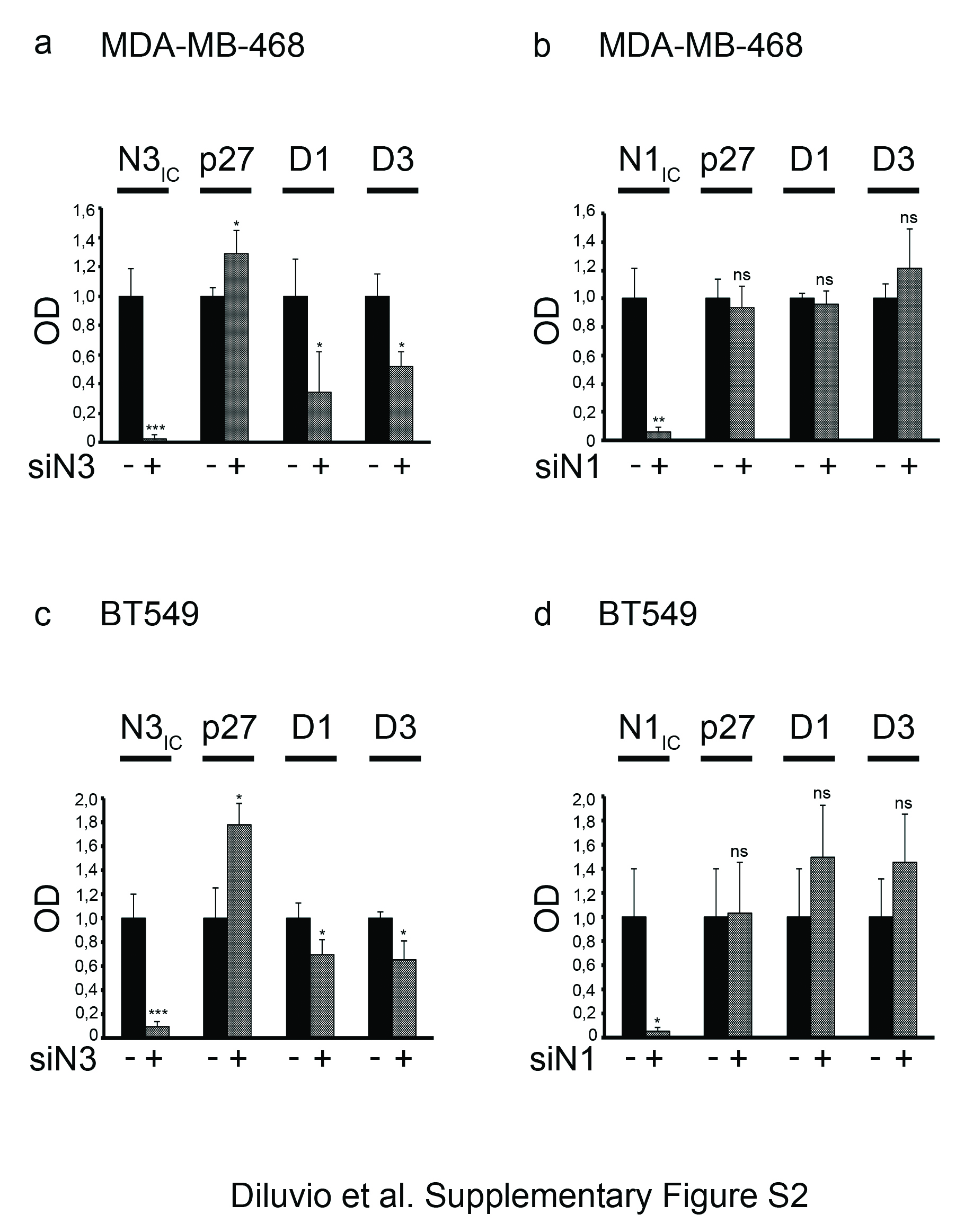

Supplement: Supplementary file 4 — Supplementary Figure S2 [file 41389_2018_51_MOESM4_ESM.jpg]

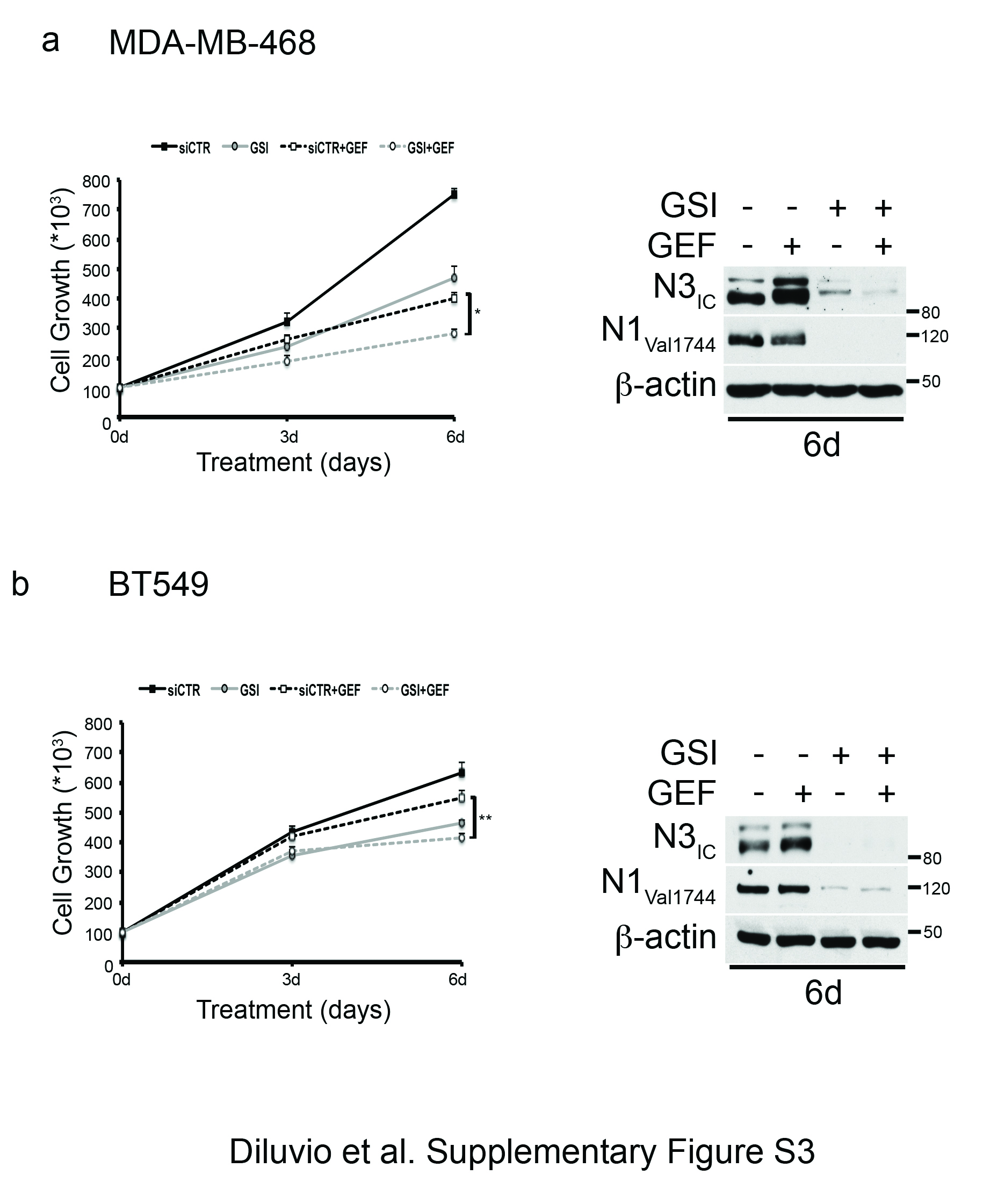

Supplement: Supplementary file 5 — Supplementary Figure S3 [file 41389_2018_51_MOESM5_ESM.jpg]

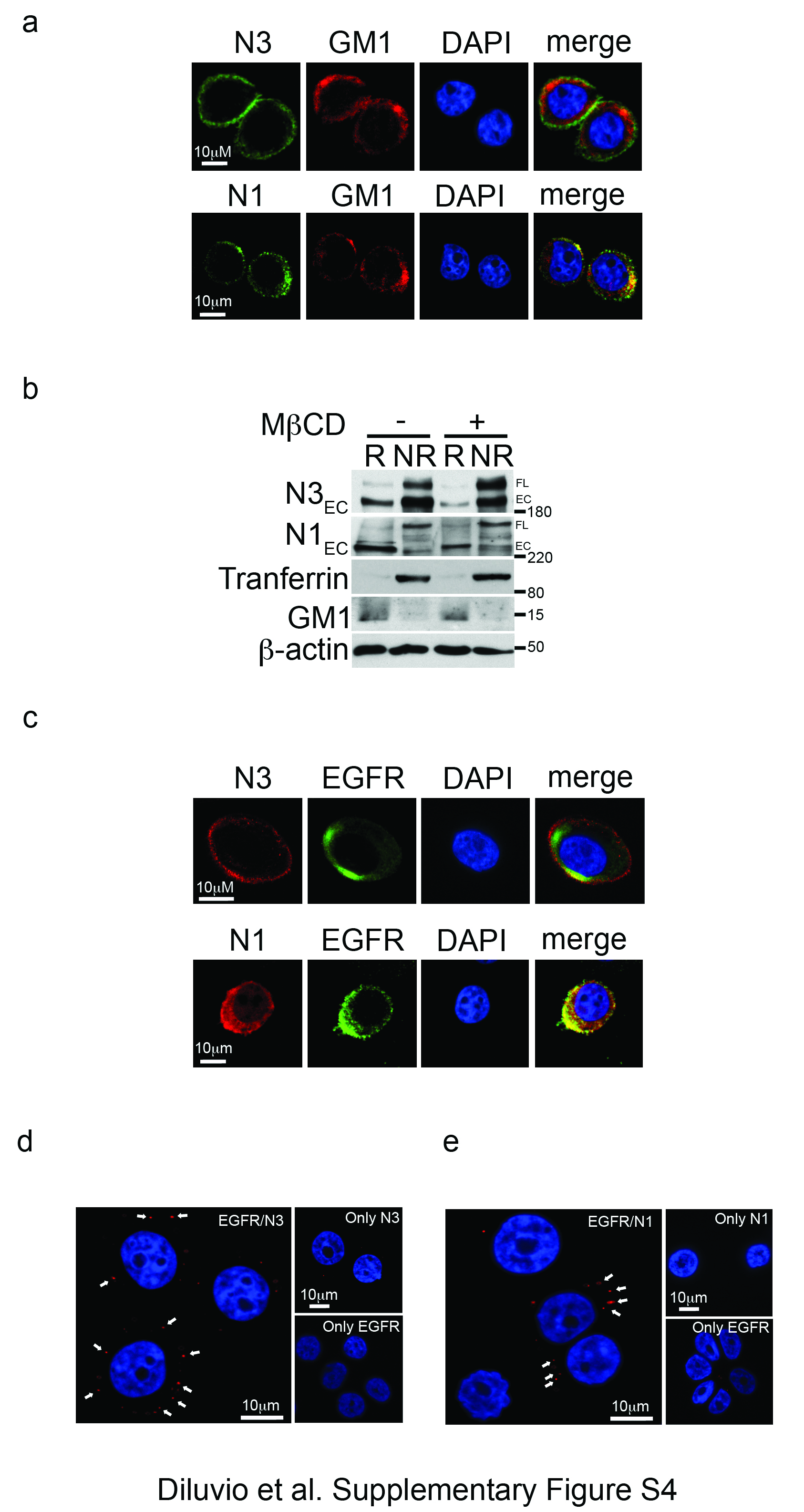

Supplement: Supplementary file 6 — Supplementary Figure S4 [file 41389_2018_51_MOESM6_ESM.jpg]
